# Relevance of Interactions between Starch-based Coatings and Plum Fruit Surfaces: A Physical-Chemical Analysis

**DOI:** 10.3390/ijms20092220

**Published:** 2019-05-06

**Authors:** Ewelina Basiak, Martin Geyer, Frédéric Debeaufort, Andrzej Lenart, Manfred Linke

**Affiliations:** 1 Department of Horticultural Engineering, Leibniz-Institute for Agricultural Engineering and Bioeconomy (ATB), Max-Eyth-Allee 100, 14469 Potsdam, Germany; mgeyer@atb-potsdam.de (M.G.); mlinke48@yahoo.de (M.L.); 2 UMR PAM 102.02 Food and Wine Physical Chemistry Lab, Univ. Bourgogne Franche-Comté, 1 Esplanade Erasme, 21000 Dijon, France; frederic.debeaufort@u-bourgogne.fr; 3 Department of BioEngineering, IUT Dijon-Auxerre, University of Burgundy, BP17867, 21078 Dijon Cedex, France; 4 Department of Food Eng. and Process Management, Faculty of Food Sciences, Warsaw University of Life Sciences-SGGW (WULS-SGGW), Nowoursynowska 159c Street, 02-786 Warsaw, Poland; andrzej_lenart@sggw.pl

**Keywords:** starch, films, coatings, epicuticular wax, surface properties, water relations

## Abstract

In order to extend the shelf life of the fruit, improve appearance, and to keep all nutrition properties of the plum from diminishing, edible coatings comprised of wheat starch and wheat starch–whey protein isolate (in ratio 80/20) were created. Stand-alone films were produced to assess properties which helped to understand the phenomena occurring on the surface level of coated plums. The properties of coatings based on starch are similar to starch coatings containing oil because the natural epicuticular wax layer of plums merges with coating materials. Adding oil doubled the contact angle value and the dispersive component of the surface tension. The workings of adhesion and cohesion, spreading coefficient, water absorption, water content, and solubility in water of the films decreased. Similar processes were observed on the fruits’ surface. In appearance, the coating process is similar to polishing the plum surface for removing crystalline wax. The color parameters of coated fruits did not significantly change. Newly formed bonds or interactions established between starch, whey proteins, water, glycerol, and oil are displayed by Fourier transform infrared (FTIR) analysis. This work revealed how the interactions between the epicuticular wax on the fruit’s surface and the hydrocolloid-based coatings affect the efficiency of the coatings.

## 1. Introduction

Biodegradable packages have a significant impact on the environment by helping to reduce waste. Total biodegradability is the highest advantage of these materials. Films and coatings can replace conventional packaging in many applications, such as disposable packaging materials, single use bags, cups, plates, containers, egg cartons, lamination coatings, etc. [1,2,3]. Preservation and protection, particularly from oxidative and microbial spoilage, extends the shelf life of many foods and their raw materials. Rapid decomposition and easy removal from the surface of fresh foods makes biodegradable and edible films and coatings very attractive for both producers and customers [3,4,5].

Hydrocolloids and lipids are two types of biomolecules generally used for the preparation of biodegradable packages. Among all of them, starch is one of the most promising materials from which to develop biodegradable films. Starch has excellent film-forming and gas barrier properties in dry conditions [6]. It also has the ability to form continuous matrices with structural complexity and thus functional diversity [7]. Another type of hydrocolloid commonly used in the production of biodegradable films is proteins. In multilayer films, layers composed of different types of polysaccharides, proteins, or lipids are able to improve the functional properties of biodegradable materials, allowing to reduce the drawback of each component (synergistic effect) than when they are used as monolayers [7,8,9,10].

Once applied onto the product surface, the coating materials are one of the food components. They can occur as single or multilayers. Coatings used to reduce transpiration losses in citrus fruits, pears, plums, and apples consist of one kind of film-forming material because the second material (wax) naturally occurs on the surface of the fruit [11,12,13].

Compared to protein-based coatings, the barrier property of starch-based materials, especially against water vapor, is less pronounced [12]. Starch exhibits a strong hydrophilic character whereas proteins have specific structures which confer a wider range of potential functional properties that compensate for the drawbacks of starch. The molecular weight of proteins is higher, so they are generally insoluble in water and thereby suited to the formation of water vapor resistant coatings and films [3,14]. However, this does not mean that bilayer or multicomponent films and coatings always have better properties than monolayers. The chemical and physical properties of coating materials depend on many fruit specific factors, such as water relations, barrier and mechanical properties, and structure of the waxy layer [15].

Plums have a natural wax on their skins [16]. This thin epicuticular wax layer, which is a complex mixture of aliphatic chain compounds (such as fatty acids, n-alkanes, alcohols, ketones, aldehydes, n-alkyl esters, and other compounds including flavonoids and pentacyclic triterpenoids), is partly permeable for water vapor and other gases [17]. The exchange of oxygen and carbon dioxide between the tissues of plums and the atmosphere takes place via pores, stomata, lenticels, and micro-cracks, so covering these structures influences further gas exchange. This explains why the storage time and shelf life of plums is limited to a few weeks [18,19,20]. A simple technology to reduce physiological activities such as transpiration and respiration is the application of edible barriers (coatings) to fruit surfaces [21]. The natural epicuticular wax layer on the plum surface (crystallized wax) by itself also contributes to extending the fruit’s shelf life [22]. However, the film-forming solutions could influence this natural wax coating, inducing changes in other properties, such as color and moisture relations [23]. Moreover, coatings influence sensory attributes such as texture, smoothness, brightness, and darkness of blue color which convince consumers to buy plums in retails.

For this work, plums were chosen for their high nutrition value, and also their relatively short shelf life. The skin can be consumed with the flesh, so plums are an excellent material for covering with edible coatings. The main objective of these investigations was to demonstrate the interaction between starch-based coatings and components of the fruit surface. Physicochemical analyses were carried out to study their effects on selected properties of the coating material. Stand-alone films were made as a model for understanding the processes which cannot be measured on the surface of the plum directly.

## 2. Results and Discussion

### 2.1. Structural and Visual Properties of Films and Coatings Applied onto the Surface of Plums

**Figure 1:**
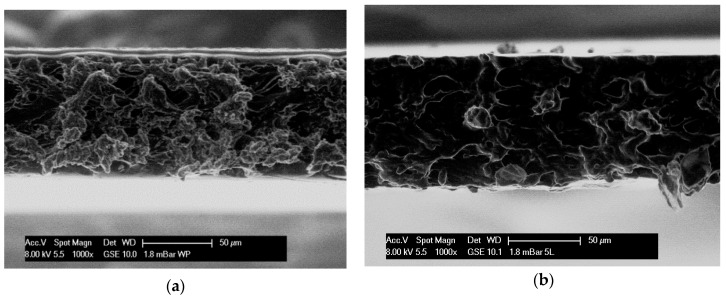

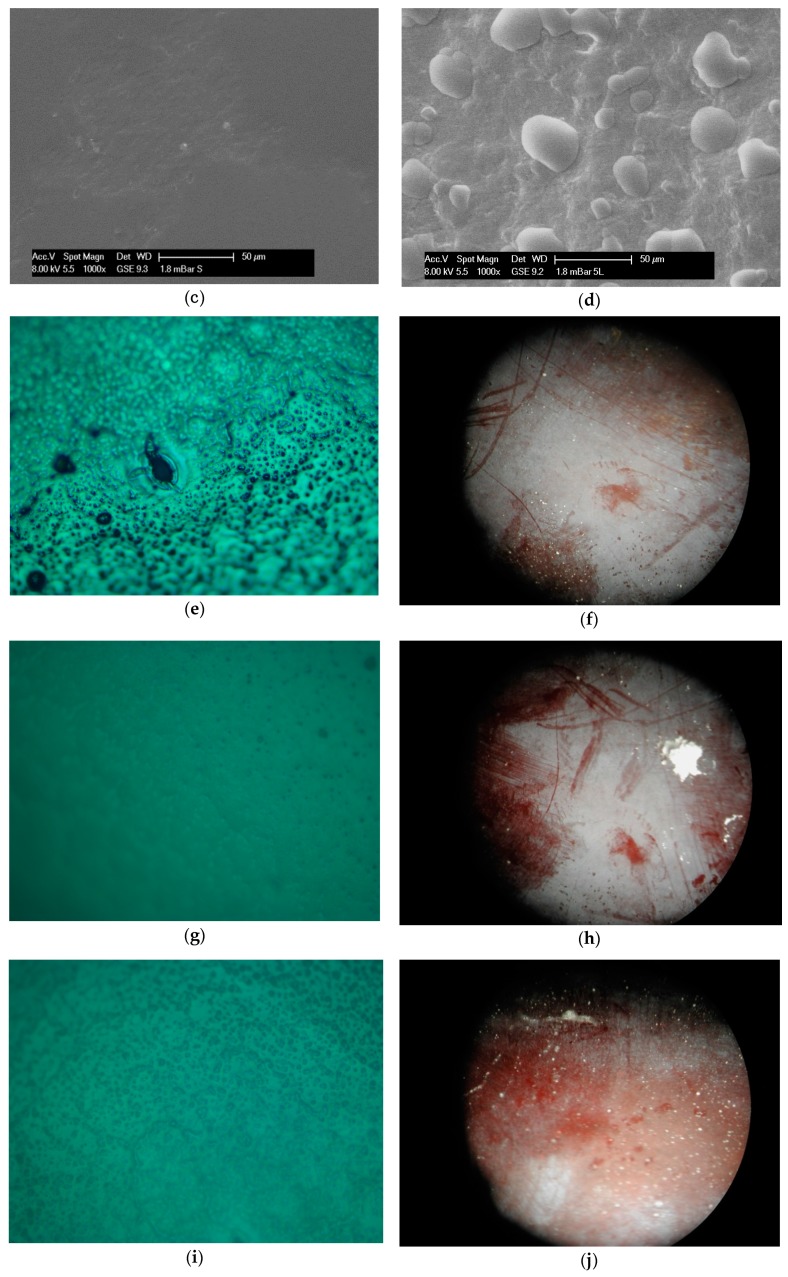
ESEM micrograph of: (**a**) cross section of starch-whey protein (80/20) films (magnification 1000×), (**b**) cross section of starch-based films (magnification 1000×), (**c**) surface exposed to air during drying of starch-based films (magnification 1000×), (**d**) surface exposed to air during drying of starch-based films with rapeseed oil addition (magnification 1000×); Polarizing microscopy image of: (**e**) surface of raw plum (magnification 200×), (**g**) plum surface after dipping in starch-based coating (magnification 200×); (**i**) plum surface after 1 h from dipping in starch-based coating (magnification 200×); Light microscopy graph of: (**f**) surface of raw plum (magnification 20×), (**h**) plum surface after dipping in starch-based coating (magnification 20×), (**j**) plum surface after 1 h from dipping in starch-based coating (magnification 20×).

The surface and the cross-section pictures achieved from environmental scanning electron microscopy (ESEM), as well as light and polarizing microscopies are presented in Figure 1. View of wheat starch and wheat starch–whey protein (80/20) films revealed a smooth, continuous, and homogenous matrix without any cracks, holes, defects etc., but did not display any significant differences between the two film types (Figure 1c). However, significant differences were observed between the cross-section micrographs (Figure 1a,b). Wheat starch contains 75% branched amylopectin chains. As such, films from pure starch are more heterogeneous and fibrillary than films with a protein isolate addition (Figure 1a,b). Even though the cross sections seem different, it does not influence the thickness of the starch and starch–whey protein films which are very similar (around 80 μm) and probably more related to dry matter content per unit area than to microstructure. After an addition of rapeseed oil, the thickness decreased to ~28μm. Rapeseed oil does not act as a plasticizer which usually entails swelling, but as a lubricant limiting the swelling (data for swelling index are not presented here). Besides fat delays, the water evaporation during the drying process, promotes the organization of a more organized matrix and fosters a densification of the structure [12]. The thickness of the starch–oil–starch coating onto plum surface is also about 28 μm.

Photographs of coated plums showed good adhesion of the starch–oil–starch films (Figure 1e–j) probably due to the natural epicuticular wax of the plum which merged with the oil present in the coating. In a previous work, [12] investigated 3-layer films derived from wheat starch (as the first layer), rapeseed oil (the second layer), and wheat starch (the third layer) (Figure 1e,f). The same structure was observed in coated plums: The skin of plum (the first layer), the wax on the skin’s surface (the second layer), and the wheat starch coating (the third layer). These layers are illustrated in Figure 1. ESEM photographs displayed that films were prepared layer by layer. This technique, however, did not result in 3-layer or multilayer structures as expected. The casting of the third layer wet the previous layers and partly solubilized the first. As such, it created an emulsion (Figure 1d). Surface images show drops of oil dispersed in the starch matrix driven to an emulsified structure, and not a multilayer one (Figure 1d). Similar effects were observed on the surface of the plum after being coated (Figure 1e–j). Plums possess a natural wax layer on their surface which is nonpolar. This kind of surface fruit could only be coated theoretically by an apolar substance. It is noteworthy that wax contains long chain and primary alcohols [24]. Film-forming solutions contain polyol-glycerol. This is probably why covering a hydrophobic surface with a hydrophilic solution is possible. It is also not excluded that starch can establish hydrophobic bonds that permit adhesion onto the wax layer, which is onto plums’ coatings. Moreover, lipid aggregates are quite small so they are properly integrated in the starch matrix as well as films. On the surface of the plum, the epicuticular wax layer is in a crystalline form (Figure 1e,f). Light and polarizing microscopic observations proved that after dipping the fruit in the film-forming solution, the wax structure was maintained (Figure 1g,h). Crystalline wax can melt, for instance, due to the temperature increase from being polished or touched, or due to being mixed with another [23]. Thus, at the beginning, crystalline wax form was visible on the surface. During the drying process, this crystalline wax layer melted with other components (Figure 1g–j) and then the coating was not visible anymore (Figure 1i,j).

Color parameters (L, a, b, ΔE, C) given in Table 1 show significant differences in value and ΔE between raw (control) and coated plums after drying (2 h). The redness parameter was 1.5 and 2 times higher for starch–whey protein (80/20) and starch coatings, respectively. Higher redness is positively perceived by consumers, which is the main visual asset. The color difference for starch–whey protein (80/20) coatings was below noticeable difference (ΔE = 1.53), while the color difference of a plum coated in starch was noticeable even to inexperienced customers (ΔE = 2.49) [25]. Data presented for films dried on Petri dishes revealed no significant color difference between them and the white control plate. On the other hand, there were significant differences between stand-alone films and coatings applied to plums. This means that both starch and starch–whey protein films (80/20) are transparent. A study [23] used two varieties of plums (*Prunus domestica*) in their experiments: cv. Hanita and cv. Ortenauer. One of the goals of the work was to compare color parameters of untreated and polished plums, where the epicuticular wax layer was removed manually. Results for color parameters obtained by these authors show that plum species played a significant role (a, b, L, ΔE). Nevertheless, L, a, and b values for untreated fruit were lower than that for cv. Jojo plums, i.e., 37.3, 2.35, −13.9, respectively. The values after taking off the epicuticular wax were even less for lightness: 28.1 and higher for redness; 2.86 for blueness; −5.0 for untreated plums of the same variety. Total color difference (ΔE) was under the value 2.3, so the slight difference in color was not visible for the human eye [26]. This fact also confirms the limited influence coatings and films have on plum color parameters. Consequently, it should not negatively affect the fruit’s acceptance by consumers. However, color chroma C parameters do not significantly differ. Untreated plums, plums coated with starch, and plums coated with starch–whey protein solutions have the same C value (no significant difference). A study [27], which measured the effect of alginate coatings on the color parameters of four plum cultivars during post-harvest storage, shows that the color chroma changed by a few units, but not significantly. The variety of the plum coated is the main indicator of color difference. Indeed, the fruit’s color chroma changed from 13 to 15 (cv. Blackamber), from 13 to 14 (cv. Larry Ann), from 44 to 45 (cv. Golden Globe), and from 43 to 45 (cv. Songold) in the first day after coating.

### 2.2. Surface Properties

Table 2 contains the data related to surface properties of starch films, starch–whey protein (80/20) films, and starch films containing oil. The contact angles and surface tension values are given in Table 2, displaying the differences between the three types of films studied. While a new surface was created, the highest disruptions of intermolecular bonds were observed for starch-based films because the starch surface is more hydrophilic than that of the starch–whey protein (80/20) matrix, and affinity to water is higher so moisturizing properties were higher. This is confirmed by the value of the contact angle which behave in opposition to the surface tension. This illustrates good wettability properties of pure starch surfaces. Biomaterials which contain oil have the most stable creation of new surfaces, so to compare pure saccharide and saccharide–protein films with saccharide–fat matrix have the highest value of contact angle and thus, the best barrier properties [12].

Scanning microscope observations revealed round drops on the surface. The same round drops of oil are visible on the starch films with rapeseed oil, and on plum surfaces a few hours after being coated in starch and starch–whey protein film-forming solutions (Figure 1d,i). So, this probably means that starch and starch–protein coatings on fruit surfaces interacted with the wax covering the plum skin, behaving like starch films with an oil addition. A few minutes after coating, polarizing photographs and light microscopy photographs revealed a homogeneous layer on the plum surface, typical of the starch solution (Figure 1g,h). Round drops related to oil appeared on the surface of the plum in the next 1 to 1.5 h. It is also noteworthy that the fruit skin displayed an apolar character after treatment, whereas the pure starch films have an almost optimal balance between dispersive and polar components. This balance was upset in starch–whey protein films. In starch films with oil addition, a dispersive component represented nearly two-thirds of the total surface tension, thus in starch and starch–whey protein films it is reduced by half.

Colocation between starch-glycerol systems and waxes explains why the formation of a coating of two opposite materials (polar and nonpolar) is possible. Moreover, surface tension is the lowest for starch films with an oil addition, which thereby means that fat causes the higher stability than is the case of wheat starch and whey protein systems. It is also confirmed by absorption. The value of starch matrices is about one-third higher than for starch–oil matrices and films with proteins. This means films with oil addition have lower water affinity than pure starch films and starch films with 20% of proteins addition [28,29,30]. Significantly, the difference between starch–oil, pure starch, and starch–whey protein films is visible in adhesion and cohesion. These parameters are higher for films without fat, so in starch and starch–protein systems, phases have a higher possibility for connecting.

**Figure 2:**
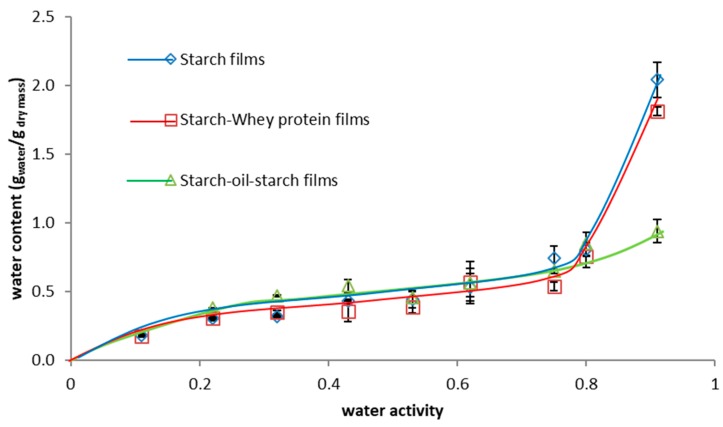
Moisture sorption isotherm of starch films, of starch- whey protein (80/20) films, and of starch-oil-starch 3-layer films (g_water_/g_dry matter_).

Looking on sorption isotherms (Figure 2), it is noteworthy that starch films sharply increase water content above a water activity of 0.60, whilst a 20% addition of protein to the structure causes water content to increase to around 0.78. Even so, slight addition of proteins as one-fifth of total dry film-forming powder mass can reduce adverse chemical, physical, and enzymatic reactions and particularly the growth of microorganisms which appeared above 0.60 of water activity (halophilic bacteria, xerophilic yeast, and filamentous fungi) [31,32]. Whey protein isolate contains hydrophobic amino acids as valine, leucine, and less hydrophobic as proline and amino acid that is part hydrophobic-lysine. As a note, 16 out of 22 protein molecules have a hydrophobic core. Whereas the hydrophobic amino acid residues are located at the core, the polar and charged amino acids favorably cover the surface of the molecule and are exposed to solvents due to their ability to create hydrogen bonds (by donating or accepting a proton from an electro-negative atom). Thus, the hydrophobic character of these amino acids reduces their affinity to water. In turn, the oil addition often reduces the water activity and all unfavorable processes above 0.8 water activity. This has a particular importance in the case of food products, such as plums, containing a lot of water and stored in high relative humidity. In the case of starch films, it was observed that water occurs (up to 0.60 of water activity) in the form of bound water. Above this value water exists as free water and hence water content is higher. The same dependence concerns starch–whey protein films, where bound water occurs up to ~78%. Fat addition reduces not only the swelling index and solubility in water but also the water content above 0.80 water activity. Hydrophobic oil drops located outside provide a more effective barrier for vapor than pure starch or starch–whey protein systems.

Table 2 gives the values of water vapor permeability (WVP). For the 0–33% relative humidity differential, the WVP of starch films and starch–whey protein (80/20) films were even ten times higher than that for starch–oil systems. In turn, for 30–75 and 30–100% of relative humidity (RH), water vapor permeability of 3-layer films (starch–oil–starch) was about half than that of starch films and starch–whey protein (80/20) films. Fat molecules acted as an effective barrier against water permeation. So even a slight addition of oil can reduce the WVP by one order of magnitude. As observed for WVP, solubility in water is significantly reduced for the films containing oil. Oxygen permeability of films is displayed in Table 2. Starch–oil emulsions are almost eight times less permeable to oxygen at 53% RH than for films without fats, and up to ten times at 75% RH. This could be a disadvantage for oil emulsions treatment for fruits because reduced oxygen permeability would induce modified atmosphere conditions with the advantage for longer shelf life of plum fruits [33,34]. Oxygen is known to be highly soluble in lipids, which explains the decrease of oxygen permeability when fat is added into the starch-based films.

### 2.3. Molecular Interactions Involved in Coatings and Films Related to Surface Properties

Fourier transform infrared (FTIR) spectroscopy is a technique which illustrates the shifting of bonds by collecting high spectral resolution data over a wide spectral range. Hence, the coatings of two opposite materials, starch–glycerol systems (or starch–whey protein–glycerol films) and waxes (polar and nonpolar), can be explained by the results obtained from infrared spectroscopy.

**Figure 3:**
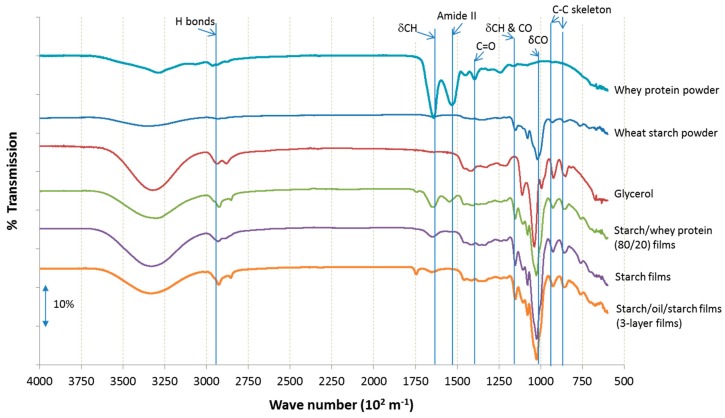
FTIR spectra of whey protein powder, of wheat starch power, of pure glycerol, of starch-whey protein (80/20) films, of starch films and of starch-oil-starch films (3-layer) at 50% RH and 25 °C.

The most important interaction between molecules which describes the wheat starch system is hydrogen bond [35]. The FTIR spectra of wheat starch powder, wheat starch–glycerol films, whey protein powder, whey protein–glycerol films, starch–oil–glycerol films, and glycerol are presented in Figure 3, which shows location and shifts due to bonding and interactions involved in matrices. Previous works [29,30,36] described spectra of starch powder and starch films with 50% of glycerol (*w*/*v*). At 3430 m^−1^ (not showed onto the graph) is located a broadband with a very strong character. OH-groups come from the absorbed starch polymer, from water, and from glycerol corresponding with this peak. Thus, broadband with wavenumbers 2928 m^−1^, 1655 m^−1^, and 1373 m^−1^ either have weak character and they were assigned with hydrogen bond, the δCH, and the C=O, respectively. Peaks with wavenumbers of 1165 m^−1^ and 1084 m^−1^ are attributed to CH and δ_CO_ groups but, in contrast to previous bonds, they have stronger energy. Additionally, a peak located at 980 m^−1^ has energy and is associated with δ_C–O_ stretching vibrations [37]. The region quite typical for saccharides is located at 1165–980 m^−1^ [38]. In turn, whey protein isolate powder and films, the wavenumber is located (not presented on the graph) at 3450 m^−1^ in the spectrum which is assigned to free N–H groups [39]. The peak located at 3293 m^−1^ in the spectrum of whey protein isolate is corresponding to hydrogen bonded N–H stretching [40]. Wavenumbers at 1700–1600 m^−1^ are characterized for amide I. So, the peak at 1643 m^−1^ also correspond to the C=O stretching vibrations of amide I [41]. The N−H stretching vibration is attributed to peak at 1565 m^−1^. Aggregated proteins show a peak assigned to 1600 m^−1^ which is characteristic of intermolecular β-sheets [42]. Peak located at wavenumber 1541 m^−1^ is attributed to the C−N stretching and N−H bending of vibrations from amide II [40,43]. Thus, C=O and N−H peaks in the amide I and II regions of whey proteins are assigned to hydrogen bonds [44]. Wavenumbers at 1400–1200 m^−1^ attributed to N−H bending and C−H stretching vibrations, it is amide III region [42]. Thus, the wavenumbers between 1250 and 1220 m^−1^ are assigned to β-sheet structures [45].

Spectrum FTIR of pure glycerol reveals bands at wavenumbers 850, 925, and 995 m^−1^ which are associated with the vibration of the skeleton C–C. The broadband at 1045 102 m^−1^ corresponds to the stretching of the C–O linkage in C1 and C3, and the peak at 1117 m^−1^ is attributed to the stretching of C–O in C2 [46].

The effect of glycerol, water, and oil can be analyzed by comparing the spectra of wheat starch powder and glycerol to the spectra of starch films of 50% glycerol and spectra of wheat starch powder, whey protein powder, and glycerol with starch–whey protein films with 50% of glycerol. Comparing wheat starch and whey protein isolate powders to the films made from 5% starch and 5% starch–whey protein (in ratio 80/20), in both matrices with 2.5% of glycerol, as seen on Figure 3, the characteristic peaks for saccharides region are increased. Bands at 1014 m^−1^ can be attributed to C–O stretching, are shifted to 1020 m^−1^ (all kinds of films). Moreover, the peak located at 2908 102 m^−1^, corresponding to C–H vibrations, also shifted to a higher position to 2932 cm^−1^ for starch and whey protein films, and to 2938 m^−1^ for the films with oil. Broadband assigned to O–H bond at wavenumber 3320 m^−1^ is shifted to 3338 m^−1^ for starch and starch–whey protein films, and to 3356 m^−1^ for oil starch systems. The addition of glycerol influences the hydrogen bonding interactions among starch and glycerol, starch–whey protein and glycerol, and starch–oil and glycerol systems. Accordingly, the decreased intensity of the peak at 3293 m^−1^ is attributed to reactions between groups of starch and NH groups of whey proteins which shift the number of NH groups [42].

## 3. Materials and Methods

### 3.1. Materials

Wheat starch was supplied by Hortimex (Konin, Poland). The whey protein isolate (WPI, ~90% protein) BiPRO was obtained from Davisco Foods International Inc. (Le Sueur, MN., USA). Anhydrous glycerol (99.9% of purity) was purchased from Sigma-Aldrich (Hamburg, Germany).

Plum fruits (*Prunus domestica* L., cv. Jojo) were harvested from the technology garden (orchard) of Leibniz-Institute for Agricultural Engineering (Potsdam-Bornim, Germany) at the commercial ripening stage and transported immediately to the laboratory. Afterwards, 180 plums (from 4 trees), with an average weight of approximately 30 grams, were split into three groups for the following treatments: The first group was the control, the second group was coated with a 5% film-forming solution of 80% wheat starch and 20% whey protein isolate, and the third group was coated with a solution of 5% of wheat starch. All fruit were weighed on a sensitive scale CPA225D (Sartorius, Göttingen, Germany) and stored in partly covered plastic trays in a climatic chamber (EMS, Hatfield, UK) under controlled environmental conditions of 5 °C, 80% relative humidity (RH), and natural convection for 1 day before undergoing the coating process.

### 3.2. Preparation of Starch and Whey Protein Edible Films and Coatings

Film-forming aqueous solutions were mixed by an Ergo Mixx blender (Bosch, Germany) and wheat starch and whey protein isolate were added in the following proportions: 100/0% and 80/20% (*w*/*w*). Glycerol was used as a plasticizer at 50% *w*/*w* of biopolymer dry weight (i.e., 33% of total dry basis). Wheat starch film-forming solutions were prepared by dissolving 5 grams of whey starch powder in 100 mL distilled water. Whey protein film-forming solutions were also prepared by 100 mL dissolving 5 grams of whey protein isolate in distilled water. The solutions were heated separately in a water bath under a 12 s^−1^, stirring at 85 °C for 30 min to denature the whey protein and to obtain complete gelatinization of the starch. Then the film forming solutions were cooled down to 40 °C. The plasticizer (2.5 g in each solution) was added and, thereafter, the solutions were mixed in the aforementioned ratios.

Obtained solutions were used for plum coatings. The fruit was immersed in the film-forming solutions for 20 s, then removed and dried for 2 hours at room conditions. From these film-forming solutions, films were prepared too (Table 3) and casted onto Petri plates (30 mL each) and fixed to obtain a consistent film thickness of about 80 µm. In order to make 3-layer films (starch-oil-starch films), only 15 mL of the starch film-forming solution was poured into the Petri dish. Then the films were dried at 25 °C and 30% RH for 48 h. To obtain a similar structure to coatings (starch coating + waxy layer of the plum fruit surface), rapeseed oil was incorporated. After drying, a layer of rapeseed oil was spread onto the dried starch layer. The layer of rapeseed oil (5 mL) was stored in the aforementioned conditions for 24 h. To form a third layer 15 mL (wheat starch film-forming solution) was applied and dried for 48 h in the same conditions. All dry films were peeled off and stored at 53 ± 1% RH and 25 ± 1 °C in desiccators containing saturated magnesium nitrate for 7 days prior to testing.

### 3.3. Coating Thickness

The mean thickness of the coating was calculated from the difference in mass between coated and uncoated fruit, the density of the coating material, and the surface area of the fruit:

(1) $$V=\frac{m}{\rho }$$

*A* = 0.809 × *FM* + 19.405 (2)

*E* = *V*/*A* (3)

where, *V* is the volume of the film-forming solution (cm^3^), *m* is the mass of the film-forming solution (g), *ρ* is the density of the film-forming solution (g cm^−3^), *e* is the mean thickness of the coating layer on each fruit (cm), *FM* is the mass of the fruit (g), *A* is the surface area (cm^2^) of the fruit.

Equation (2) was established from preliminary experiments. Thirty-six plums were scanned using a camera supported by a 3D ScanBook scan system along with ScanWare Enterprise 3.8 software (Scanbull, Hameln, Germany). The measured surface area was set into relation to fruit mass. This relation found by a regression analysis is valid only in the range between 30–80 g of fruit mass.

The relevant mass of film-forming solution covering the fruit surface was determined from the difference in weight of the plums before and after applying the coating (dipping).

### 3.4. Film Thickness

Film thickness was measured with a PosiTector 6000 (DeFelsko, Ogdensburg, NY, USA) digital micrometer to the nearest 1 µm in 0–100 µm range and to the nearest 5 µm in the 100–1000 µm range. Prior to film thickness measurements, the electronic gauge was calibrated at 74 and 139 µm using standards to be close to the thicknesses of the samples. The thickness of each film was measured at five points at the center of each film and at four points around the perimeter. An average value was used in the calculations. At least ten replications of each formulation were made.

### 3.5. Surface Properties Measurements

#### 3.5.1. Contact Angle Determination

Contact angle (θ) is the angle described as a relationship between the surface tension and one of three phases: Liquid phase L, solid phase S, or vapor phase V according to the Young equation [47]. The contact angle was measured by the sessile drop method: A droplet (about 1.5 µL) of a test liquid was placed on a horizontal film surface (in stand-alone films). Measurements were done using a DGD-DX goniometer equipped with the DIGIDROP image analysis software (GBX, Romans-sur-Isere, France), according to the methodology of [48]. The contact angle was measured on both sides of the drop and averaged. The contact angle and drop volume measurements were carried out over 120 s. The effect of evaporation was assessed on an aluminum foil considered as an impermeable reference surface and subtracted from the sample. Then, the rate of evaporation was considered in the study of the kinetics of wetting and absorption [49]. Measurements for all samples were done on the side of the films exposed to air while drying in order to prevent the support (Petri dish) effect. Measurements were taken of a minimum of ten samples of each film recipe.

#### 3.5.2. Critical Surface Tension

The surface tension of the liquid tested (γL) was measured by the sessile drop method and Laplace–Young approximation [50,51]. The estimation of the critical surface tension (γC) of the starch-based films was obtained using the Zisman method [52]. The critical surface tension (γc) value of the film has been obtained from the extrapolation the linear regression of the cos θ of various liquids according their surface tension (γL). Extrapolation at cos θ = 1 yields the value of the critical surface tension of the film. Cyclopentanol, diiodomethane, ethylene glycol, glycol, methyl benzoate, n-octane, polyethylene glycol, tetradecane, water, and 1-bromonaphtalane were selected as the liquids for which the surface tension properties, dispersive, and polar components are known, and which are also used for the determination of the surface tension and its components. Ten repetitions, at least, were done on each film formulation.

#### 3.5.3. Surface Tension

The surface tension, or surface free energy ($\gamma_{S}\gamma_{S}$), and its dispersive ($\gamma_{S}^{D}\gamma_{S}^{D}$) and polar ($\gamma_{S}^{P}\gamma_{S}^{P}$) components were calculated using the Owens–Wendt method [53] as evident in Equations (4) and (5):

(4) $$\gamma_{S}=\gamma_{S\;}^{P}+\gamma_{S}^{D}\gamma_{S}=\gamma_{S}^{P}+\gamma_{S}^{D}$$

(5) $$\gamma_{L}=\left(1+\cos\theta \right)=2\;\times \;\left(\sqrt{\gamma_{L}^{P}\gamma_{S}^{P}}+\sqrt{\gamma_{L}^{D}\gamma_{S}^{D}}\right)\gamma_{L}=\left(1+\cos\theta \right)=2\;\times \;\left(\sqrt{\gamma_{L}^{P}\gamma_{S}^{P}}+\sqrt{\gamma_{L}^{D}\gamma_{S}^{D}}\right)$$

In Equation (4), appear two unknowns $\gamma_{S}^{P}\gamma_{S}^{P}$ and $\gamma_{S}^{D}\gamma_{S}^{D}$, so it is insufficient to determine the surface free energy of a polymer. The contact angle was measured using at least two liquids, knowing their respective surface tensions and their dispersive $\gamma_{S}^{D}\gamma_{S}^{D}$ and polar $\gamma_{S}^{P}\gamma_{S}^{P}$ components. Water and diiodomethane were used for the determination of surface tensions components. Measurements were made at least ten times.

#### 3.5.4. Adhesion, Cohesion, and Spreading Coefficient

Work of adhesion (per unit area, Wa), work of cohesion (per unit area, Wc), and spreading coefficient (for a liquid over a solid, Ws) were calculated using [54] equations:

(6) $$W_{a}=W_{a}^{P}W_{a}^{P}\;+\;W_{a}^{D}W_{a}^{D}\;↔2\left(\sqrt{\gamma_{L}^{P}\gamma_{S}^{P}}+\sqrt{\gamma_{L}^{D}\gamma_{S}^{D}}\right)2\left(\sqrt{\gamma_{L}^{P}\gamma_{S}^{P}}+\sqrt{\gamma_{L}^{D}\gamma_{S}^{D}}\right)\;=\;\gamma_{L}\left(1+\cos\theta \right)\gamma_{L}\left(1+\cos\theta \right)$$

(7) $$W_{c}=2\gamma_{\mathrm{LV}}\gamma_{\mathrm{LV}}$$

(8) $$W_{s}=W_{a}-W_{c}=\gamma_{\mathrm{SV}}-\gamma_{\mathrm{LV}}-\gamma_{\mathrm{LS}}\gamma_{\mathrm{SV}}-\gamma_{\mathrm{LV}}-\gamma_{\mathrm{LS}}$$

### 3.6. Transport and Solubility Properties

#### 3.6.1. Water Content of Films

The water content was measured in samples of 2 × 2 cm, by determination of the weight loss of the film after drying at 105 °C for 24 h in a drying oven UT20 (Thermo Scientific Heraeus, Hanau, Germany), and is expressed as gram of water per gram of dry matter. All experiments were performed in triplicate.

#### 3.6.2. Absorption Index

The absorption index was measured to assess the impact of immersion on water absorption. The samples were prepared from five different films of the same kind. They were cut into 2 × 2 cm pieces and weighed on an analytical balance CPA224S-OCE (Sartorius, Göttingen, Germany) with an accuracy of 0.0001. They were then immersed in distilled water (25 °C) for 2 min. Wet samples were wiped with filter paper to remove excess liquid before weighing. The amount of adsorbed water was calculated in percentages. The measurement was repeated for each type of film three times, and the average was taken as the final result.

#### 3.6.3. Solubility in Water

Water solubility was determined according the [55] method. Films were cut into 2 × 2 cm pieces and dried at 105 °C for 24 h in drying oven UT20 (Thermo Scientific Heraeus, Hanau, Germany) before weighing. Films were individually placed in 50 mL beakers filled with 20 mL of distilled water, capped and stored at 25 ± 1 °C for 24 h. Film pieces were then taken out and dried at 105 °C for 24 h to determine the final weight of the dry matter. These steps were repeated three times. Loss of total soluble matter was calculated from the initial and final dry weight of the films.

#### 3.6.4. Moisture Sorption Isotherm

The sorption isotherm of films was determined at 25 °C. Samples of films were cut into small pieces (2 × 2 cm) and weighed to the nearest 0.0001 g in preweighed vials. Films were stored up to equilibrium in ten desiccators, each containing a saturated salt solution which fixed the relative humidity. A wide range of RH was selected: Lithium chloride (11%), potassium acetate (22%), magnesium chloride (33%), potassium carbonate (43%), magnesium nitrate (53%), sodium nitrite (65%), sodium chloride (75%), ammonium sulfate (81%), and ammonium dihydrogen phosphate (93%).

Film samples were weighted periodically, up to the equilibrium was reached and checked up to 9 months. The final (equilibrium) water content was measured by drying the films at 105 °C for 24 h. The amount of water absorbed is expressed as grams of water per gram of dry matter. Measurements were done in triplicate for each film recipe.

### 3.7. Water Vapour (WVP) and Oxygen (OP) Permeabilities of Films

The water vapor permeability of the films was measured gravimetrically according to [26] who adapted the [56] standard method to hydrophilic edible films and coatings. Film samples were placed between two rubber rings on top of glass cells containing silica gel and sodium chloride or distilled water, allowing to obtain internal RH of the permeation cells at ~0%, 75%, and 100%. The permeation cells were then placed in a ventilated climatic chamber KBF 240 (Binder, Tuttlingen, Germany) set on a RH of 30% and a temperature of 25 °C, and weight was recorded daily for at least 10 days. Water vapor permeability (gm^−1^ s^−1^ Pa^−1^) was calculated using the following equation:

(9) $$\mathrm{WVP}=\frac{\Delta m\cdot e}{A\cdot \Delta t\cdot \Delta p}$$

where, Δ*m*/Δ*t* is the moisture loss per unit of time (g s^−1^); *A* is the film area exposed to moisture transfer (8.04 × 10^−4^ m^2^); *e* is the film thickness (m); and, Δ*p* is the water vapor pressure differential between the two sides of the film (Pa). Measurements were performed at least three times for each differential tested.

The oxygen permeability was measured using the manometric method according the [57] standard using a GDP-C (Brugger, München Germany). The test chambers of the permeation cell were first degassed under vacuum, then the upper side was swept by a humidified oxygen flow at a rate of about 1.33 mL s^−1^ at atmospheric pressure. The increase in pressure in the downside chamber during the test period was assessed and displayed by an external computer. Data was recorded and permeance was calculated by GDP-C software (with temperature compensation). The sample temperature (25 °C) was adjusted using an external thermostat Haake F3 with water bath K (Thermo Fisher Scientific, Karlsruhe, Germany). The desired RH was regulated in an external saturation system (53% and 75%), so humidified oxygen gas circulated in the permeation cell.

### 3.8. Color Parameters

The color of the films was determined using a colorimeter CR-300 (Minolta, Japan) using the CIE LAB color parameters: L, from black (0) to white (100); a, from green (−) to red (+); and b, from blue (−) to yellow (+) [58]. The color of the films was expressed as the total color difference (Δ*E*) and color chroma according to the following equations [59]:

(10) $$\Delta E=\sqrt{{\left(L-L^{*}\right)}^{2}+{\left(a-a^{*}\right)}^{2}+{\left(b-b^{*}\right)}^{2}}$$

(11) $$C=\sqrt{a^{2}+b^{2}}C=\sqrt{a^{2}+b^{2}}$$

where, L*, a*, b*, and *C* are the color parameters of a white standard used as the film background (L* = 96.74, a* = 0.09, b* = 2.20).

Color parameters of the plum skin were determined individually using six fruits of each replicate (measured at three points on the surface of each fruit), using a colorimeter CM-2600d (Minolta Europe, Germany). Three determinations were performed.

### 3.9. Microstructure Observations

Film microstructure was observed using an environmental scanning electron microscope ESEM XL-30 (Philips, Japan). A 0.5 × 1.0 cm film was fixed on the support using double-sided adhesive, at an angle of 90° to the film surface which allowed the observation of the cross section of the film. All the film samples were cut with a new razor blade to limit morphological damage. Films were focused up to 15000×, and magnifications ranging from 800x to 8000x were selected, with an intensity of 8 kV and absolute pressure of 230 Pa in presence of water (RH ~30% at 5 °C). No special preparation, such as palladium or gold coating, was necessary for ESEM observation.

### 3.10. Microscopy Observations

Film structure was observed using the light microscope (Vision Engineering, Emmering, Germany) with coupled camera (Nikon Coolpix 990, Japan). The uncoated plum skin, the skin of plums immediately after dipping, and the skin of plums coated and dried were focused up to 400x, and magnifications ranging from 10× to 40× were selected. No special preparation was necessary for microscopic observation.

Film microstructure was also observed using a polarizing microscope BX51 (Olympus, Japan). Therefore, small sections of plum skin (approximately 1.0 × 0.5 cm) were cut off with a sharp razor blade. One untreated skin sample was observed directly and two other samples were coated by dipping the skin into a solution of starch film-forming solution. Coated plum skin was observed directly and after air drying. For light microscopic analysis, the samples were placed on microscopic slides and illuminated from the top side with an external light source (100 Watt gooseneck lamp). For epifluorescence images, an Olympus U-MBFL3 reflected light brightfield mirror cube equipped with a built-in neutral density filter was used. Excitation was performed with an Exfo X-Cite 120Q lamp. Images were taken at magnifications of 1000×, 2000× and 4000×.

### 3.11. Fourier Transform Infrared with Attenuated Total Reflection (FTIR-ATR)

The Fourier transform infrared spectra from each film were obtained using a spectrometer IFS 28 (Bruker, Ettlingen, Germany) by means of attenuated total reflectance (ATR) using ZnSe crystal. Samples were cut from self-standing films by shape razor and scanned. All the spectra had an average of 64 scans at a resolution of 4 × 10^2^ m^−1^, from 650 × 10^2^ to 4000 × 10^2^ m^−1^, and determined at 25 °C on films equilibrated at 53% RH. This analysis aimed at determining the modifications at the molecular scale of the surface induced by the different coating component addition.

### 3.12. Statistical Analysis

All experiments were prepared using completely randomized designs. Statistical analysis was performed with Statgraphics Plus, version 5.0 (Manugistics Corp., Rockville, MD, USA). The analysis of variance (ANOVA) and Fisher’s least significant difference (LSD) multiple comparisons were performed to detect significant differences in the properties of the films. The significance level used was 0.05.

## 4. Conclusions

The natural wax structures existing on the plum surface can act as a component of the starch-based coating. By alcohol groups, epicuticular crystal wax merges with the components of the starch–glycerol film-forming solution. Hence, starch and starch–whey protein coatings have the same properties as starch–oil films and probably starch–whey protein (80/20)–oil systems. ESEM and light microscopy confirmed that layered structures were combined. The epicuticular wax crystal coating was not visible few hours after coating, but still exists, dispersed in the starch–glycerol–water system. As such, a 3-layer structure did not appear on the surface of the fruit as expected, but a thin (~28μm) emulsion was evident instead. Resistance to oxygen and water vapor permeability was improved. To summarize, observation and quantitative data illustrate a positive relationship between the presence of a starch or starch–whey protein coating and the shelf life of plums.

Further studies should focus on the key problem of how these findings can be used to extend the shelf life of the fruits. The weightings of the individual coating components have to be varied so that a maximum keeping quality of the fruits can be ascertained. In addition, research has to take into account several additional aspects such as consumer acceptability and costs of the coating material.

## Figures and Tables

**Table 1:** Colour parameters of the surface of non-coated plums (control), of starch films, starch-whey protein (80/20) films, starch coated plums, of starch-whey protein (80/20) coated plums. Thicknesses of films and coatings were similar (about 28 × 10^−6^ m).

Surface	L	a	b	ΔE	C
Uncoated plums (control)	54.43 ± 4.90 ^a^	2.23 ± 0.37 ^b^	−8.18 ± 1.73 ^a^	-	8.48 ^b^
Starch film	96.20 ± 0.31 ^b^	0.32 ± 0.10 ^a^	3.72 ± 0.61 ^b^	1.52 ^a^	4.02 ^a^
Starch-whey protein (80/20) films	95.13 ± 0.32 ^b^	0.46 ± 0.11 ^a^	4.36 ± 0.73 ^b^	2.59 ^b^	5.08 ^a^
Starch coated plums	54.45 ± 11.04 ^a^	4.61 ± 1.37 ^c^	−8.93 ± 3.31 ^a^	2.49 ^b^	10.04 ^b^
Starch-whey protein (80/20) coated plums	54.01 ± 11.42 ^a^	3.64 ± 1.21 ^c^	−7.76 ± 3.34 ^a^	1.53 ^a^	8.57 ^b^

^a,b,c^ Values having the same letter for a parameter are not significantly different at *p* level < 0.05.

**Table 2:** Physico-chemical characteristics of films: surface tension of liquids (γ_L_), surface tension of films (γ_S_) and their dispersive (γ_S_^D^) and polar (γ_S_^P^) components, adhesion (W_A_), cohesion (W_C_) and spreading coefficients (W_S_), swelling index, water content, solubility in water, water vapour and oxygen permeabilities at 25 °C.

Film Characteristics		Film Composition			
		Starch-Whey Protein (80/20)		Starch	Starch-Oil-Starch (3-layer)
**Thickness (µm)**		79.7 ± 11.2 ^b^		80.8 ± 12.59 ^b^	27.7 ± 5.69 ^a^
Surface properties	Contact angle (^o^)	Water (γ_L_ = 72.8 mNm^−^^1^)	61 ^b^	43 ^a^	77 ^c^
	Surface free energy (m N m^−1^)	γ_S_^D^	38.0 ^a,b^	35.4 ^a^	40.6 ^b^
		γ_S_^P^	22.9 ^b^	24.9 ^b^	15.5 ^a^
		γ_S_	60.9 ^b^	60.3 ^b^	56.1 ^a^
	W_A_ (mJ m^−2^)	125.86 ^b^		126.80 ^b^	115.12 ^a^
	W_C_ (mJ m^−2^)	121.80 ^b^		120.56 ^b^	112.12 ^a^
	W_S_ (mJ m^−2^)	−4.06 ^a^		−6.24 ^b^	−3.00 ^a^
Transport and solubility properties	Swelling index (%)	53.49 ± 1.86 ^c^		39.20 ± 1.43 ^b^	34.91 ± 1.39 ^a^
	Water content (kg _water_ kg^−1^_dm_)	2.04 ± 0.05 ^a^		3.24 ± 0.50 ^c^	2.69 ± 0.21 ^b,c^
	Solubility in water (%)	13.06 ± 0.18 ^b^		19.67 ± 0.17 ^c^	10.70 ± 0.72 ^a^
	Water vapour permeability (10^−^^10^ g m^−^^1^ s^−^^1^ Pa^−1^)	33–0% RH	4.99 ± 0.06 ^b^	5.24 ± 0.26 ^b^	0.57 ±0.46 ^a^
		75–30% RH	6.37 ± 1.03 ^c^	7.70 ± 0.85 ^c^	3.55 ± 2.80 ^b^
		100–30% RH	7.16 ± 0.54 ^c^	7.87 ± 0.65 ^c^	3.40 ± 0.31 ^b^
	Oxygen permeability (10^−^^14^ cm^3^ m^−^^1^ s^−^^1^ Pa^−^^1^)	53% RH	7.44 ± 1.07 ^c^	7.23 ± 1.00 ^c^	0.96 ± 0.02 ^a^
		75% RH	11.69 ± 0.86 ^d^	7.41 ± 1.39 ^c^	1.12 ± 0.05 ^b^

^a^^,b,c^ Values having the same letter for a parameter are not significantly different at *p* level < 0.05.

**Table 3:** Composition of film-forming solution (100g) used for both plum coating or film making.

Film	Wheat Starch (S)	Whey Protein Isolate (WPI)	Rapeseed Oil (O)	Glycerol	Water
	(g)	(g)	(g)	(g)	(g)
Starch	5	0	0	2.5	95
starch-whey proteins (80/20)	4	1	0	2.5	95
Starch-oil-starch (3-layer)	5	0	3	2.5	95
